# Glioblastoma invasion factor ODZ1 is induced by microenvironmental signals through activation of a Stat3-dependent transcriptional pathway

**DOI:** 10.1038/s41598-021-95753-6

**Published:** 2021-08-10

**Authors:** Veronica Vidal, Olga Gutierrez, Ana Talamillo, Carlos Velasquez, Jose L. Fernandez-Luna

**Affiliations:** 1 Genetics Unit, Hospital Universitario Marqués de Valdecilla and Instituto de Investigación Marqués de Valdecilla (IDIVAL), Avda. Valdecilla s/n, 39008 Santander, Spain; 2 Neurosurgery Service, Hospital Universitario Marqués de Valdecilla and Instituto de Investigación Marqués de Valdecilla (IDIVAL), 39008 Santander, Spain

**Keywords:** Cancer, Neuroscience, Cell signalling

## Abstract

We have previously shown that the transmembrane protein ODZ1 serves for glioblastoma (GBM) cells to invade the surrounding tissue through activation of RhoA/ROCK pathway. However, the transcriptional machinery used by GBM cells to regulate the expression of ODZ1 is unknown. Here we show that interaction with tumor microenvironment elements, mainly activated monocytes through IL-6 secretion, and the extracellular matrix protein fibronectin, induces the Stat3 transcriptional pathway and upregulates ODZ1 which results in GBM cell migration. This signaling route is abrogated by blocking the IL-6 receptor, inhibiting Jak kinases or knocking down Stat3. Furthermore, we have identified a Stat3 responsive element in the ODZ1 gene promoter, about 1 kb from the transcription start site. Luciferase-reporter assays confirmed that the promoter responds to the presence of monocytic cells and this activation is greatly reduced when the Stat3 site is mutated or following treatment with a neutralizing anti-IL-6 receptor antibody or transfecting GBM cells with a dominant negative variant of Stat3. Overall, we show that monocyte-secreted IL-6 and the extracellular matrix protein fibronectin activate the axis Stat3-ODZ1 and promote migration of GBM cells. This is the first described transcriptional mechanism used by tumor cells to promote the expression of the invasion factor ODZ1.

## Introduction

ODZ1 belongs to a family of large transmembrane proteins that have been mainly associated with embryonal development of the central nervous system^1^. Recently, we described the expression of ODZ1 in glioblastoma (GBM) primary cells derived from surgical specimens^2^ and showed that overexpression of the full length protein or the intracellular domain increased the invasion capacity of these tumor cells both in vitro and in vivo. Consistently, blockade of ODZ1 by deleting the intracellular domain or knocking down its expression drastically reduced invasiveness of GBM cells. ODZ1 has been related to different tumors, including B3 thymoma, through a chromosomal translocation involving exon 2 of C11orf73 and intron 19 of ODZ1. The fusion transcript is supposed to trigger nonsense-mediated decay resulting in mRNA degradation^3^, although no experimental confirmation was presented. ODZ1 has also been located close to the integration site of hepatitis B virus into the genome of hepatocellular carcinoma^4^ but functional consequences of this gene fusion have not been explored. Finally, a naturally occurring deletion of the entire ODZ1 gene has been described in a patient with GBM^2^. Tumor cells derived from this patient have a deficiency in cytoskeletal organization. Therefore, they are unable to form actin-dependent protrusions and have lower migration and invasion capacities. Invasiveness is a key hallmark of GBM hindering effective therapy and causing tumor recurrence, generally in close proximity to the original tumor site^5^. However, attempts to target the mechanisms involved in GBM invasiveness including cytoskeletal reorganization, cell migration and degradation of the extracelular matrix have not been translated into useful therapeutic strategies thus far^5,6^. One way to widen the options of therapeutic intervention is to fully understand the interactions between GBM cells and their microenvironment, including immune cells, secreted cytokines or growth factors and molecules of the extracelular matrix. Among the immune cells, bone marrow-derived marophages and resident microglia represent an important cell population comprising 30–50% of all cells in GBM tumors^7^. These cells secrete a number of cytokines and chemokines that may be used by GBM cells to promote cell growth, invasion or immunosuppression^8^ through activation of intracelular pathways. To this end, one of the main signaling mediators in tumor cells is Stat3, which is involved in driving cell survival, proliferation, invasion and metastasis among other activities^9,10^ and this transcription factor is tipically activated by triggering growth factor and cytokine receptors. Recently, it has been established a gene signature that stratifies GBM patients into Stat3-high and Stat3-low cohorts and showed that inhibition of Stat3 signaling reduces Stat3-high cell viability and tumorigenicity in vivo^11^.

In the present study, we showed that GBM cells activate a Stat3-dependent upregulation of ODZ1 in the presence of monocytic cells. This activation depends on the secretion of IL-6 by monocytic cells and it can also be induced by fibronectin, a ubiquitous extracellular matrix component. Moreover, we found an active Stat site in the ODZ1 promoter that responds to both stimuli and showed that mutagenesis of this site or blocking interaction of IL-6 with its cognate receptor abrogates transactivation.

## Results

### Expression of ODZ1 correlates with activation of Stat3

**Figure 1:**
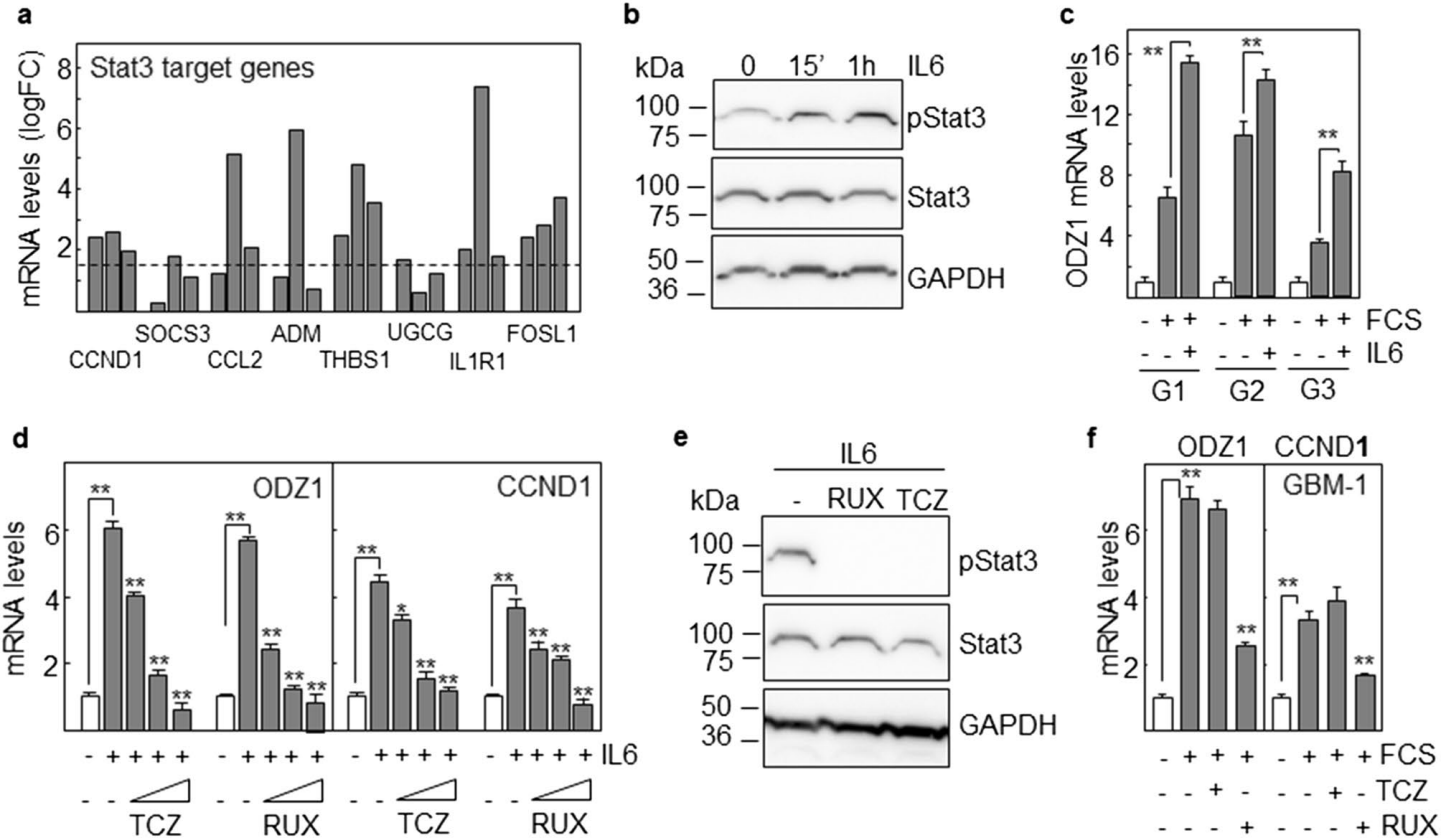
Stat3 target genes and ODZ1 are expressed by GBM cells in response to FCS and IL-6. (**a**) Expression of Stat3 target genes were analyzed in a gene expression microarray previously described^12^. Bars represent fold-change (FC) in gene expression of GBM cells incubated with 10% FCS relative to cells without FCS in three different cultures. Differences were selected as significant using a logFC cut-off of 1.5 (dotted line). (**b**) Western blot analysis confirmed that exposure of GBM cells to IL-6 activated Stat3 by inducing phosphorylation at Ser 727. Stat3 and GAPDH were used to assure equal loading. (**c**) Three different GBM primary cell lines were cultured in the presence of FCS with or without IL-6 and the mRNA levels of ODZ1 were determined after 24 h by qPCR. (**d**) GBM cells were incubated with IL-6 in the presence of increasing concentrations of the blocking anti-IL-6 receptor antibody Tocilizumab (TCZ) (100, 200 and 400 ng/ml) or the Jak-Stat pathway inhibitor Ruxolitinib (RUX) (5, 10 and 30 μM) and the expression of ODZ1 and CCND1 was quantitated by qPCR. (**e**) Western blot analysis showing a reduced phosphorylation of Stat3 in the presence of RUX (30 μM) and TCZ (400 ng/ml). Stat3 and GAPDH were used to assure equal loading. (**f**) GBM cells were incubated with FCS in the presence of inhibitors and the expression of ODZ1 and CCND1 was assessed by qPCR. Histograms represent the mean of three independent experiments ± S.D. Asterisks represent significant differences (**p < 0.01).

We have previously described that ODZ1 is induced upon differentiation of stem-like GBM cells in the presence of fetal calf serum (FCS)^2^. We first reanalyzed a gene expression array comparing stem-like GBM cells in the absence or presence of FCS^12^ and found an increase in the mRNA levels of at least eight genes known to be Stat3 targets^13^ including CCND1, SOCS3, CCL2, ADM, THBS1, UGCG, IL1R1 and FOSL1 (Fig. 1a). Stat3 can be phosphorylated and translocated to the nucleus in the presence of FCS^14^ but this transcription factor is mostly activated by the cytokine IL-6. Therefore, we explored this in our system by culturing GBM cells in the presence of IL-6 at different time periods (Fig. 1b). As early as 15 min after exposure to IL-6 there was phosphorylation of Stat3 that further increased by 1 h in the presence of the cytokine. Both FCS and IL-6 upregulated the mRNA levels of ODZ1 in three independent GBM primary cell cultures derived from surgical specimens (Fig. 1c). Moreover, in the presence of FCS addition of IL-6 further increased the expression of ODZ1. In order to confirm that upregulation of ODZ1 was produced through binding of IL-6 to its cognate receptor, we cultured GBM cells in the presence of IL-6 and increasing concentrations of Tocilizumab, an anti-IL-6 receptor antibody that blocked binding to IL-6^15^. The neutralizing antibody efficiently inhibited the IL-6-induced expression of both ODZ1 and the Stat3 target gene CCND1 in a dose-dependent manner (Fig. 1d). Similar results were obtained when cells were treated with increasing concentrations of Ruxolitinib, a Jak-Stat signaling inhibitor used in the clinic (Fig. 1d). Both treatments efficiently inhibited the phosphorylation of Stat3 at the highest concentration (Fig. 1e). Ruxolitinib also significantly reduced the expression of both ODZ1 and CCND1 in response to FCS (Fig. 1f). Since current commercial antibodies against ODZ1 are not suitable for western blotting, at least in our hands, ODZ1 expression was determined at the level of mRNA by quantitative RT-PCR (qPCR).

**Figure 2:**
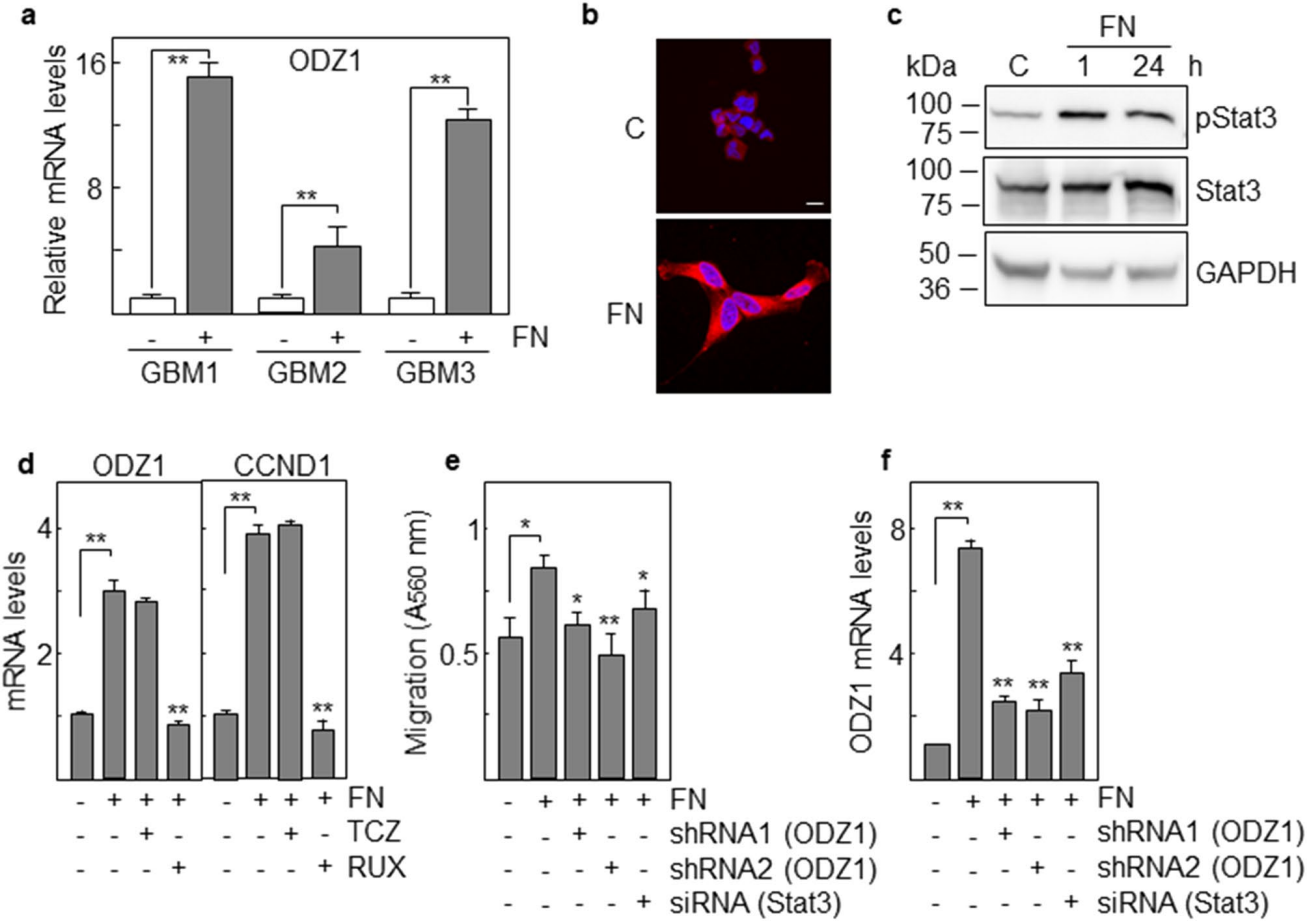
Fibronectin induces migration of GBM cells through Stat3-mediated expression of ODZ1. (**a**) GBM primary cell lines were cultured on a fibronectin (FN)-coated surface and three days later cells were analyzed for the expression of ODZ1 mRNA by qPCR. (**b**) GBM1 cells were cultured as above and then immunolabeled with anti-ODZ1 antibodies and the signal visualized by immunofluorescence. Samples were counterstained with DAPI to label nuclei. Scale bar: 5 μm. (**c**) Western blot analysis of phosphorylated Stat3 in GBM1 cells cultured in the presence of FN for 1 and 24 h. Stat3 and GAPDH were used to assure equal loading. (**d**) GBM cells were incubated with fibronectin (FN) in the presence of the inhibitors TCZ and RUX and the expression of ODZ1 and CCND1 was assessed by qPCR. (**e**) GBM1 cells transfected with two different ODZ1-specific shRNAs or a mixture of Stat3-specific siRNAs were cultured on FN and their migration capacity was determined using a Boyden-like system. (**f**) Cells cultured as described in (**e**) were analyzed for the expression of ODZ1 by quantitative PCR. Irrelevant shRNA or siRNAs (−) were used as negative controls. Histograms represent the mean of three independent experiments ± S.D. *p < 0.05, **p < 0.01.

Fibronectin is a main component of the GBM extracellular matrix and it has been shown to trigger the Stat3 pathway^16^. Thus, we cultured three GBM primary cell lines in fibronectin-coated plates and found an increase in the mRNA levels of ODZ1 (Fig. 2a) and the protein expression of ODZ1 by immunofluorescence (Fig. 2b) that correlated with activation of Stat3 as determined by upregulation of the phosphorylated form of this transcription factor (Fig. 2c). Fibronectin-dependent upregulation of both ODZ1 and CCND1 was blocked by the Jak-Stat inhibitor Ruxolitinib (Fig. 2d). Since ODZ1 is able to function as an invasion factor, we studied the contribution of ODZ1 to the migration of GBM cells in response to fibronectin. In the presence of fibronectin GBM cells increased their migration capacity in a Boyden chamber-like assay (Fig. 2e). However, knockdown of ODZ1 by using two ODZ1-specific shRNAs or a mixture of Stat3-specific siRNAs (Fig. 2f) significantly reduced migration of GBM cells in response to fibronectin (Fig. 2e).

### ODZ1 expression is induced in GBM cells by monocytic cells

**Figure 3:**
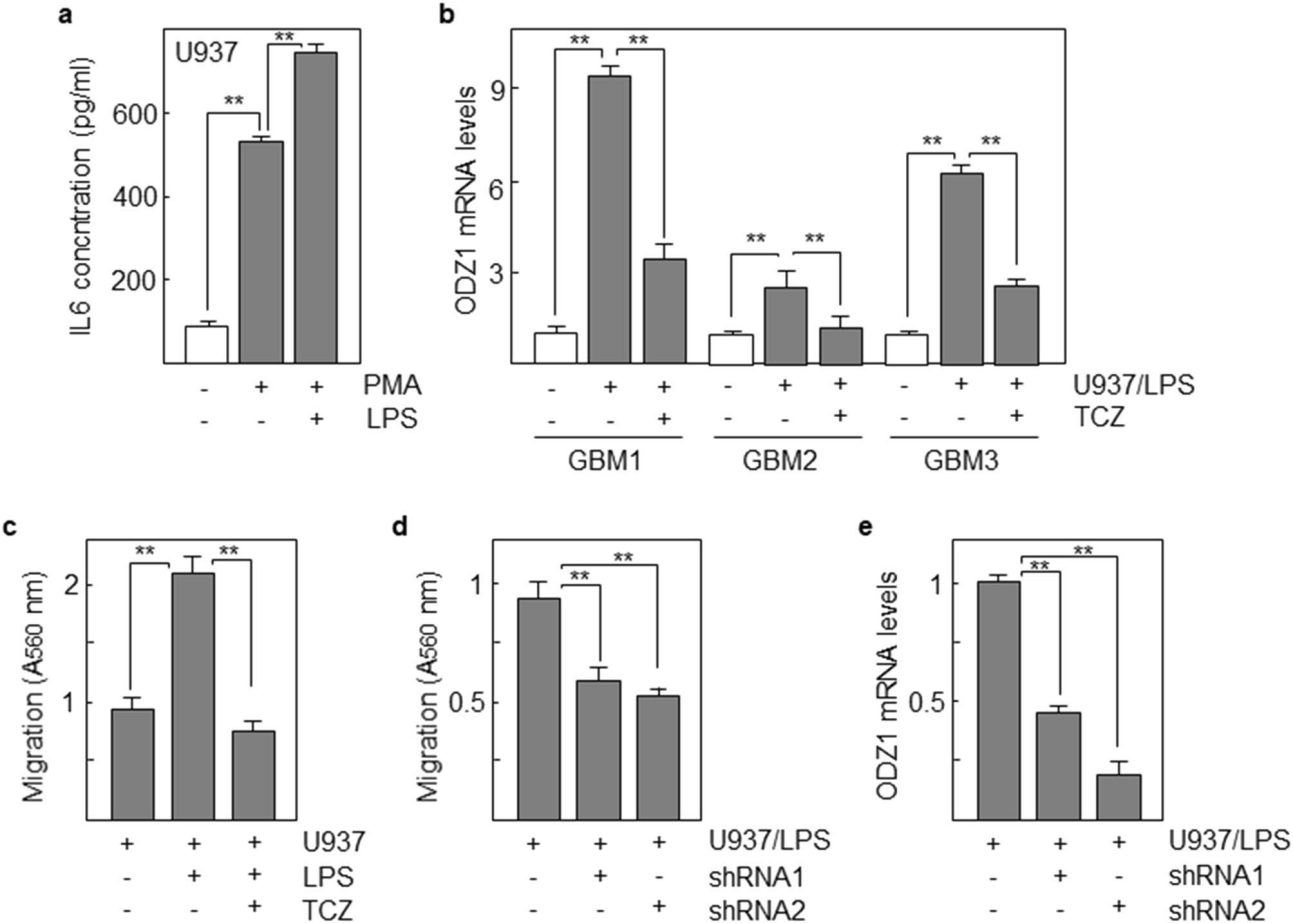
Monocytic cells promote upregulation of ODZ1 in GBM cells. (**a**) U937 monocytic cells were incubated in the presence of the differentiation promoter PMA with or without lipopolysaccharide (LPS) as a monocyte/macrophage activator. Supernatant were analyzed 48 h later for the presence of soluble IL-6 by using a specific immunoassay. (**b**) Expression of ODZ1 mRNA in three GBM cell lines derived from different patients after coculture with activated (LPS-treated) monocytes in the presence or in the absence of 200 ng/ml Tocilizumab (TCZ). (**c**) Migration assay of GBM1 cells cultured with or without LPS and TCZ. (**d**) Migration assay of GBM1 cells transfected with two ODZ1-specific shRNAs. (**e**) Downregulation of ODZ1 mRNA levels in GBM1 cells transfected with ODZ1-specific shRNAs. An irrelevant shRNA (−) was used as negative control. Histograms represent the mean of three independent experiments ± S.D. **p < 0.01.

GBM tumors are infiltrated by monocytic cells that differentiate to macrophages within the tumor tissue. Monocytes/macrophages are a common source of a number of cytokines, including IL-6. We used a monocytic cell line, U937 in coculture experiments to study the effect of activated monocytes on the expression of ODZ1. First we confirmed that U937 cells secreted quantifiable amounts of IL-6 into the culture medium that were highly increased (about fivefold) following differentiation of U937 towards macrophages in the presence of phorbol diester PMA (Fig. 3a). Moreover, after activation with lipopolysaccharide (LPS), the secreted levels of IL-6 were even higher. Coculture of three different GBM primary cell lines with activated macrophage-like U937 cells induced a high increase (about 3- to 9-fold) in the mRNA levels of ODZ1. Consistent with our previous results, the expression of ODZ1 was drastically reduced in the presence of neutralizing anti-IL-6 receptor antibody (Fig. 3b). The migratory capacity of GBM cells was also explored in this experimental model. LPS-activated U937 increased the migration of GBM cells, that was abrogated when Tocilizumab was added to the culture (Fig. 3c). Similar results in reducing GBM cell migration in the presence of activated U937 were obtained by knocking down the expression of ODZ1 with two different shRNAs (Fig. 3d,e).

**Figure 4:**
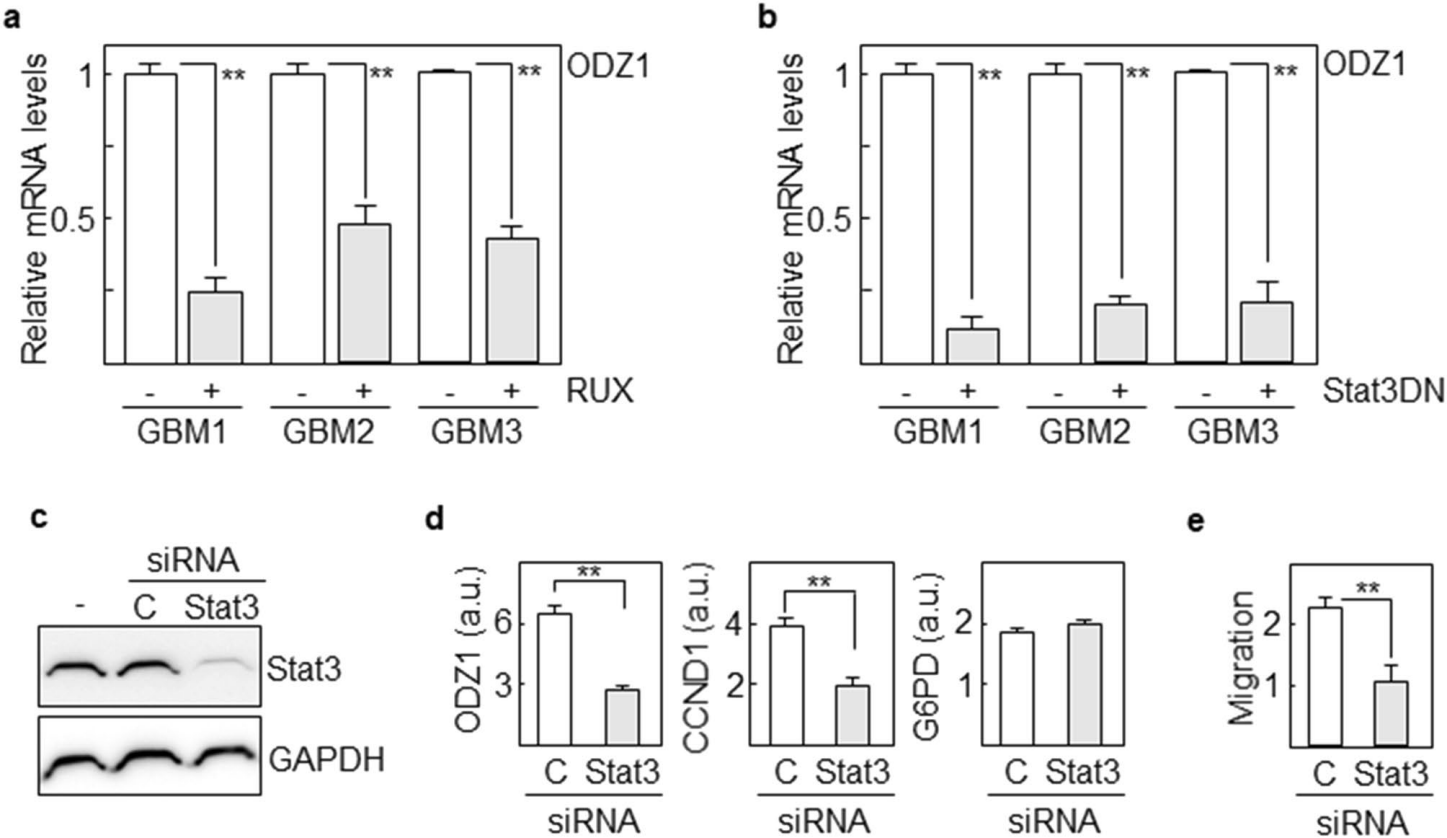
Upregulation of ODZ1 in GBM cells cocultured with U937 is abolished by blocking the IL-6 receptor–Stat3 pathway. (**a**) qPCR analyses of ODZ1 levels in GBM cell lines cocultured with activated U937 in the presence of the Jak-Stat signaling inhibitor, Ruxolitinib (RUX). (**b**) ODZ1 levels in GBM cells transfected with a dominant-negative variant of Stat3 following coculture with activated U937. Empty vector was used as negative control (−). (**c**) Western blot analysis showing downregulation of Stat3 protein levels in cells transfected with specific siRNAs. C, irrelevant siRNAs. GAPDH were used to assure equal loading. (**d**) ODZ1 levels were determined in GBM1 cells transfected with a Stat3-specific siRNA mix after coculture with activated U937 cells. CCND1 and G6PD were used as positive and negative controls for Stat3 target genes respectively. (**e**) Migration assay of siRNA transfected GBM cells cocultured with U937. Migration was determined by measuring absorbance at 560 nm in a spectrophotometer. Irrelevant siRNAs were used as negative control (C). Histograms represent the mean of three independent experiments ± S.D. **p < 0.01.

In order to explore whether upregulation of ODZ1 in GBM cells was due to activation of Stat3 through monocytic cells, we cocultured activated U937 cells and GBM primary cell lines with or without Ruxolitinib. Following coculture for 48 h, the expression of ODZ1 mRNA was clearly reduced by more than half in all three cell lines treated with the inhibitor (Fig. 4a). The role of Stat3 in ODZ1 gene expression was further confirmed by transfecting GBM cell lines with a splice variant of Stat3 that lacks the transactivation domain and blocks Stat3-mediated gene expression in a dominant negative manner. Blockade of Stat3 activity efficiently downregulated the expression of ODZ1 in all GBM cell lines in the presence of activated U937 cells (Fig. 4b). Moreover, we knocked down Stat3 in GBM cells with a mixture of Stat3-specific siRNAs prior to culture with activated monocytic cells. Downregulation of the protein levels of Stat3 (Fig. 4c) was consistent with a reduction in the expression levels of ODZ1 mRNA (more than 2-fold) and the Stat3 target gene CCND1 (about 2-fold) but not the negative control G6PD (Fig. 4d). Additionally, the migratory capacity of GBM cells was also reduced (Fig. 4e).

### The ODZ1 promoter contains a Stat3 site that responds to IL-6 and coculture with U937

**Figure 5:**
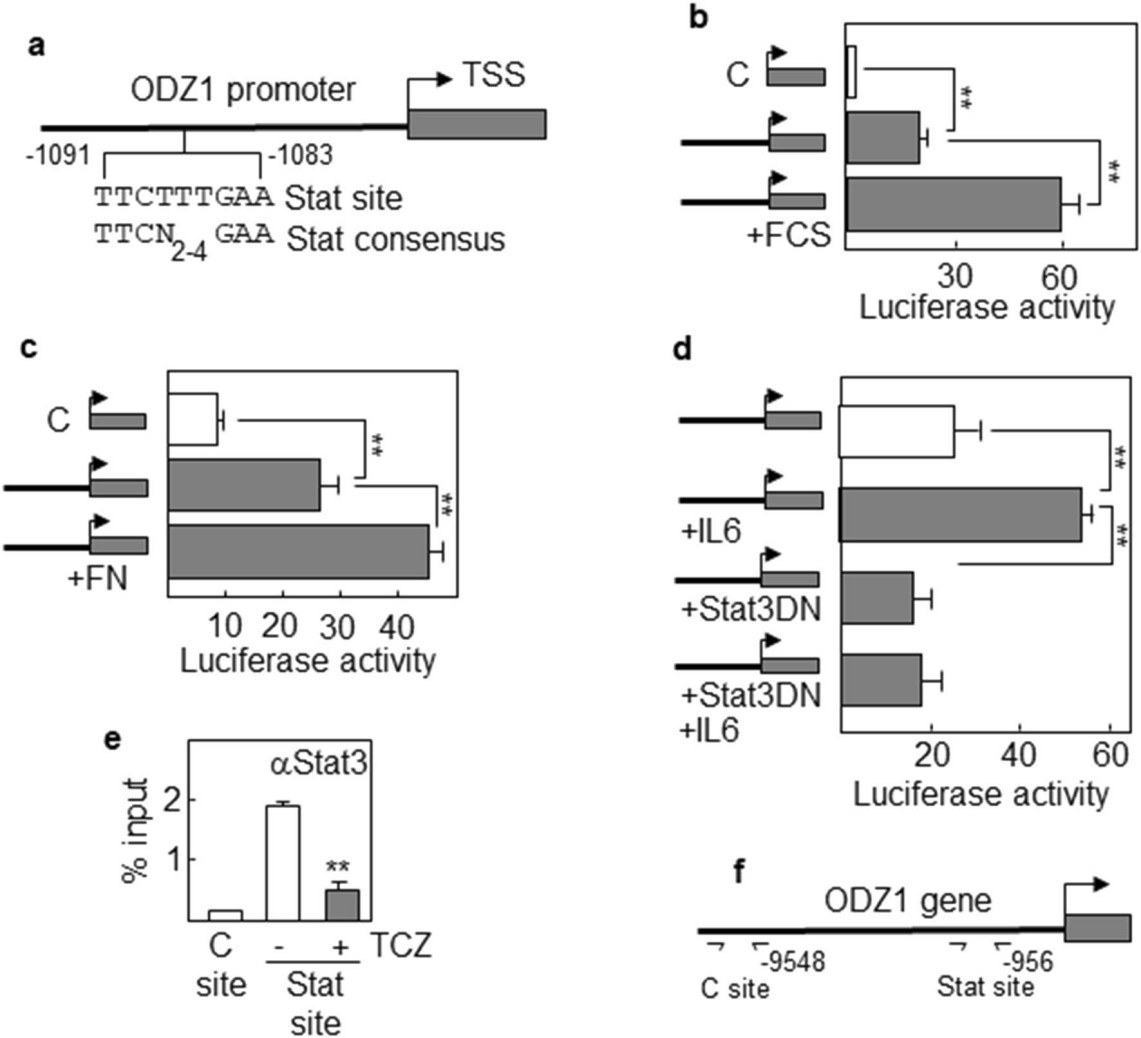
ODZ1 promoter responds to Stat3-activating stimuli. (**a**) Schematic representation of ODZ1 promoter, indicating the position and sequence of the Stat site relative to the transcription start site (TSS). Stat consensus sequence is included for comparison. GBM1 cells transfected with an ODZ1 promoter-luciferase reporter construct were incubated with FCS (**b**) or cultured onto fibronectin-coated surface (**c**) and luciferase activity was used as an indicator of promoter activity in response to the stimuli. (**d**) GBM1 cells were cotransfected with the reporter construct and a dominant negative variant of Stat3 and the response of ODZ1 promoter to IL-6 was determined by analyzing luciferase activity. (**e**) Chromatin immunoprecipitation assay using GBM cells incubated in the presence of IL-6 with or without Tocilizumab. Immunoprecipitated DNA was analyzed by qPCR with primers flanking the Stat site in the ODZ1 promoter or an irrelevant control site further upstream. (**f**) Schematic representation of the ODZ1 gene showing target sites for ChIP assay. Histograms represent the mean of three independent experiments ± S.D. **p < 0.01.

We found a Stat consensus site in the ODZ1 promoter about 1 kb from the transcription start site (Fig. 5a). The promoter was cloned into a luciferase reporter vector and the construct was introduced in GBM cells by nucleofection. Consistent with our previous results, culturing GBM cells with FCS activated the ODZ1 promoter (3-fold) after 48 h of exposure (Fig. 5b). Similarly, fibronectin induced a significant activation of the promoter although at a lower level (Fig. 5c). Additionally, the Stat3-activating cytokine IL-6 induced (more than 2-fold) activation of the ODZ1 promoter that was completely abrogated when cells were transfected with the dominant negative variant of Stat3 (Fig. 5d). The specific binding of Stat3 to the ODZ1 promoter was assessed by a chromatin immunoprecipitation assay in cells treated with IL-6. Anti-Stat3-immunoprecipitated DNA was amplified with primers flanking the Stat site and primers flanking a region more than 9 kb further upstream used as a control site (Fig. 5d,e). An amplification signal was only detected in the region containing the Stat site, which was highly reduced (about 4-fold) in the presence of Tocilizumab (Fig. 5e).

**Figure 6:**
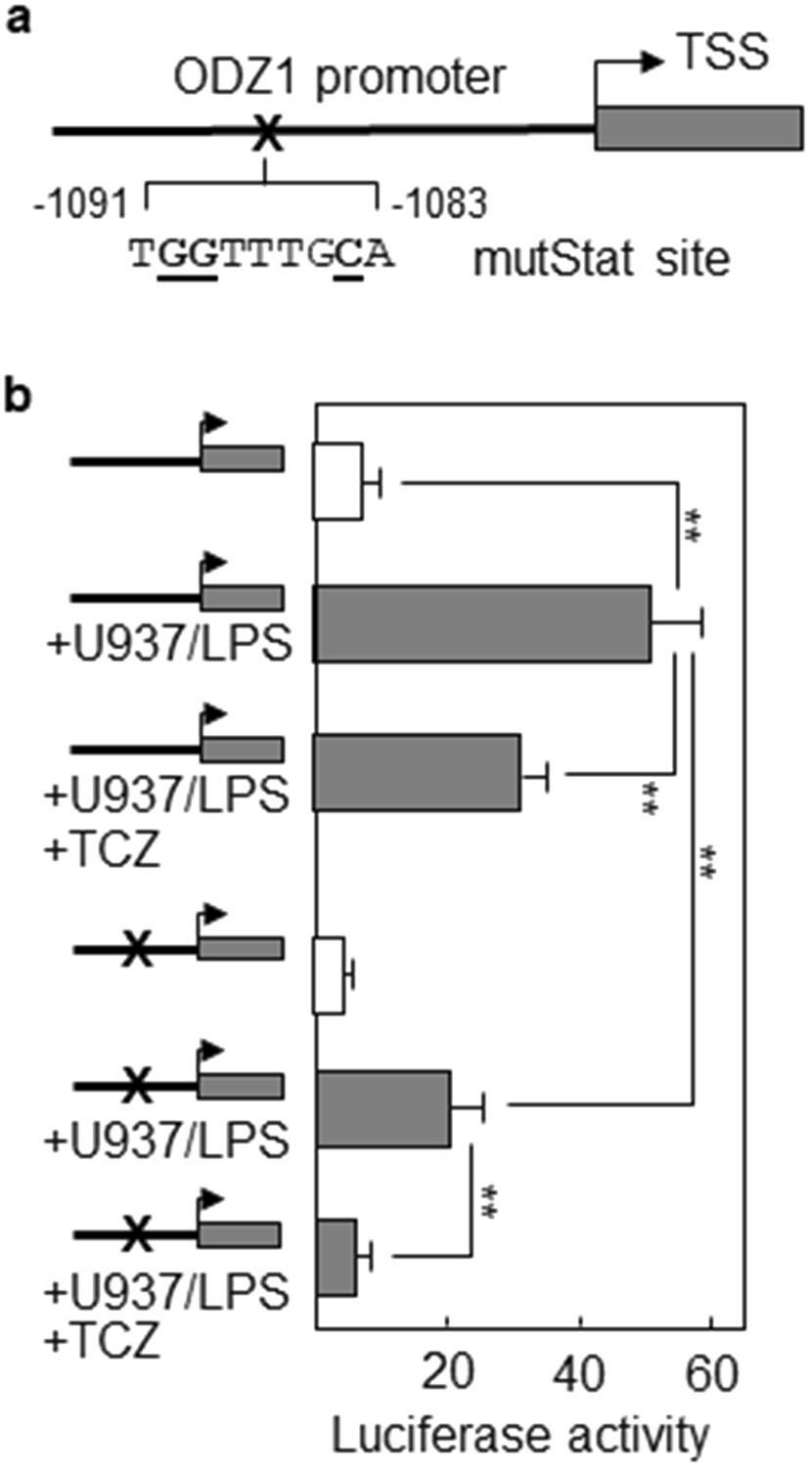
ODZ1 promoter responds to Stat3-activating stimuli. (**a**) Schematic representation of ODZ1 promoter, indicating the nucleotides within the Stat site changed (bold and underlined) by site-directed mutagenesis. (**b**) Wild type or mutant ODZ1 promoter were introduced into GBM cells and response of the promoter to the presence of activated U937 cells with or without Tocilizumab (TCZ) was determined by analyzing luciferase activity. Histograms represent the mean of three independent experiments ± S.D. **p < 0.01.

To confirm the involvement of the Stat3 consensus site in the activation of the ODZ1 promoter, we changed three key nucleotides of the Stat-binding sequence by site-directed mutagenesis (Fig. 6a). LPS-activated U937 greatly induced promoter activity (7-fold) that was reduced in the presence of the IL-6 receptor blocking antibody, confirming that secretion of IL-6 by monocytic cells is a relevant mechanism for ODZ1 promoter activation (Fig. 6b). Moreover, mutagenesis of the Stat site drastically reduced (2.5-fold) activation of the promoter by U937 when GBM cells were transfected with the mutant promoter. This activity was further reduced to a basal (no activation) level in the presence of Tocilizumab (Fig. 6b).

## Discussion

We have previously described that ODZ1 is an important factor for GBM cells to invade the surrounding tissue^2^. A key feature of GBM is hypoxia of the tumor core due to the rapid growth of this aggressive glioma, and we showed that hypoxic environments induces the expression of ODZ1 gene by epigenetic modification of its promoter^17^. The complex tumor microenvironment includes extracellular matrix, secreted factors that promote cell signaling and tumor-associated cells. Among these, monocytes/macrophages that infiltrate the tumor constitute a relevant cell population that mediate cell–cell communications with the tumor^18^. Based on this, we argued that some of these microenvironmental interactions may serve to induce the expression of ODZ1, which facilitates GBM cells to invade the neighboring parenchyma. We showed that fetal calf serum, fibronectin and IL-6 were able to upregulate ODZ1 in GBM cells. All these stimuli are known to activate the Jak-Stat transcriptional pathway in tumor cells^14,16,19^. Downregulation of this pathway by blocking binding of IL-6 to its cognate receptor or inhibiting Jak kinase activity reduced ODZ1 expression in response to IL-6 but, as expected, only the kinase inhibitor impeded upregulation of ODZ1 in cells incubated with fibronectin or fetal calf serum. Stat3 is a master regulator of mesenchymal transformation in GBM^20^ and it has been described that Stat3 can be constitutively activated in GBM cells, which facilitates tumor growth and regulates the immune microenvironment^21^. We also showed that coculture of GBM cells with IL-6-secreting monocytic U937 cells induces the expression of ODZ1 in GBM cells and their migration, and both are blocked by Tocilizumab, a neutralizing antibody against the IL-6 receptor or knocking down Stat3 expression with specific siRNAs. In line with this, IL-6 has been detected to be abnormally elevated in the cerebrospinal fluid and within the tumor of GBM patients^22,23^. In cancer, increased levels of IL-6 result in increased activation of Jak-Stat3 pathway, which correlates with poor prognosis^24^. Under this scenario, we searched for Stat3 sites within the ODZ1 promoter and found a Stat consensus sequence about 1 kb from the transcription start site. Moreover, a promoter-containing reporter construct was activated in GBM cells by monocytic U937 cells. Very recently, we described the presence of a CpG site at the 3′ end of the ODZ1 promoter that was hypomethylated in GBM cells^17^. The promoter activity was reduced in response to hypoxia following mutagenesis of the CpG site. Similarly, we mutated the Stat site in the ODZ1 promoter and showed that the response to U937 was drastically reduced. The role of the IL-6-Stat3-ODZ1 axis was further confirmed by using the neutralizing IL-6 receptor antibody which decreased the promoter activity in GBM cells cocultured with U937 cells but also the activity of the mutated promoter. Since no more Stat binding sites have been found in the first 3 kb upstream from the transcription start site of the ODZ1 gene, these data indicate that Stat-independent pathways may have a role in the ODZ1 transcriptional response to IL-6 as it also happens in other biological models^25^. A recent work stratified GBM patients into Stat3-high and Stat3-low cohorts and showed that Stat3 inhibitors were effective in reducing Stat3-high tumor cell viability and tumorigenicity in mouse xenograft models^11^. Interestingly, the use of Stat3 inhibitors also reduced the invasive potential of Stat3-high GBM cells. We previously demonstrated that knockdown of ODZ1 gene expression or removal of the intracellular region of the protein resulted in a drastic reduction of the invasion capacity of GBM cells both in vivo and in vitro^2^. A naturally occurring deletion of the entire ODZ1 gene has been described in primary tumor cells obtained from a GBM patient. These cells were unable to reorganize the cytoskeleton for cell migration after stimulation with FCS and maintained a rounded morphology with no or short protrusions^2^. Consistently, here we showed that inhibition of Jak-Stat pathway in GBM cells reduces the expression of ODZ1 and promotes cell migration.

**Figure 7:**
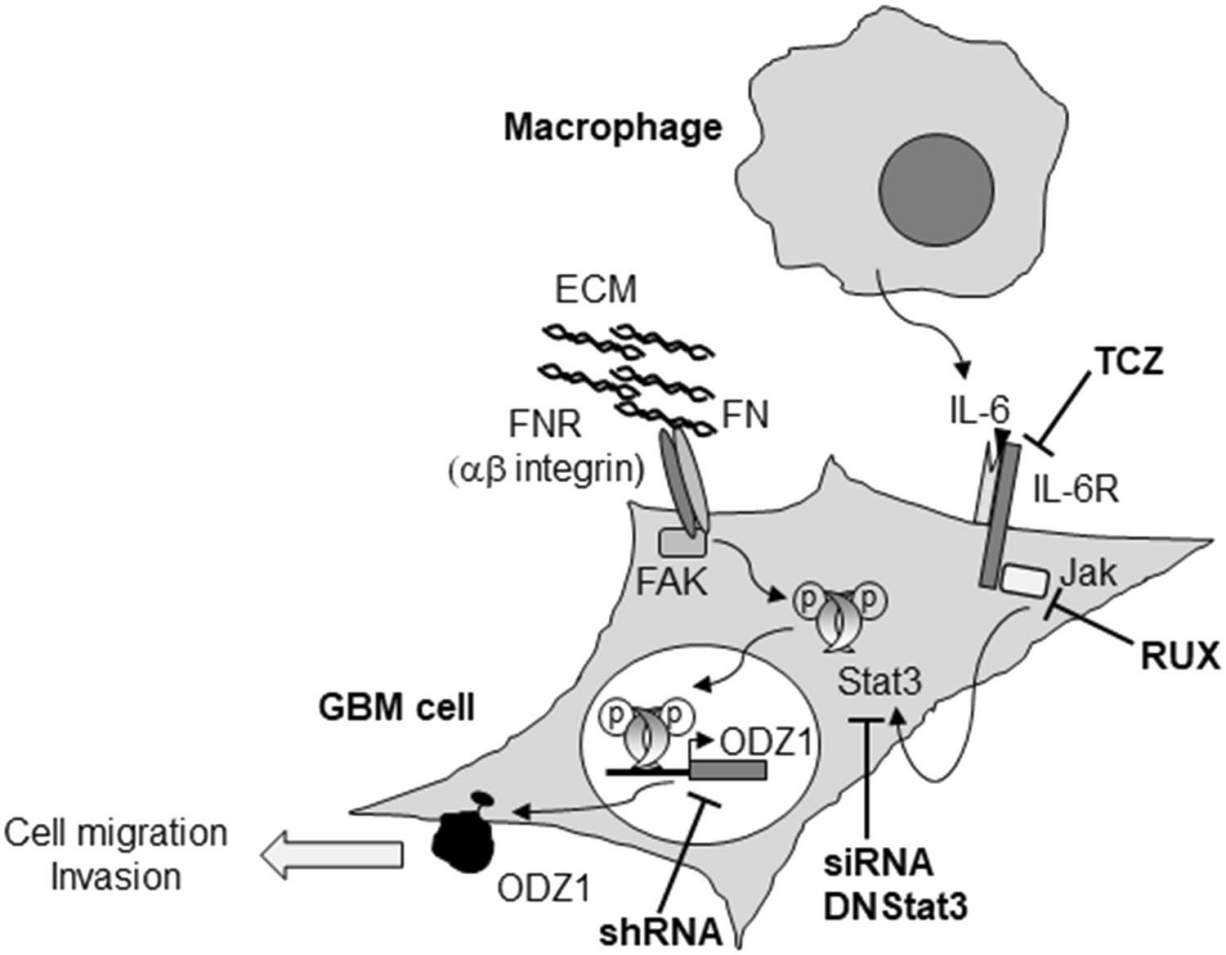
Schematic representation of the Stat3-ODZ1 transcriptional pathway. Both IL-6 secreted from activated monocytes/macrophages, present in tumor microenvironment, and fibronectin, as part of the extracellular matrix, are able to trigger phosphorylation-mediated activation of Stat3 which binds to the promoter of ODZ1 and induces its expression. Strategies used here to block the Stat3-ODZ1 pathway are indicated. *TCZ* Tocilizumab, *RUX* Ruxolitinib.

Overall, the results presented here show that two key components of GBM microenvironment, fibronectin and IL-6-secreting monocytic cells, induce activation of Stat3 mediated by receptor-associated kinases, tipically FAK for the fibronectin receptor^26^ and Jak for the IL-6 receptor^27^. Activated Stat3 binds to a consensus sequence in the ODZ1 promoter and transactivates ODZ1 gene (Fig. 7). This expression pathway contributes to GBM cell migration and links with our previous mechanistic data about the function of ODZ1^2^. Thus, fibronectin/IL-6-Stat3-ODZ1 signaling strengthen current data on the relevance of Stat3 inhibition in GBM patients and highlights potential targets of this pathway. However, ODZ1 which contributes to GBM invasiveness^2^, a key feature of this aggressive cancer, is likely to be activated by different transcriptional pathways in response to an array of cell migration stimuli in the microenvironment of GBM cells. Further studies will shed more light on alternative or complementary molecular mechanisms that contribute to the expression of ODZ1.

## Materials and methods

### Cell cultures

IDH1/2 wild type primary GBM cell lines used in this study were previously established from surgical specimens in our laboratory^12^. Tumor cells were maintained as neurospheres in serum-free DMEM/F12 medium (Invitrogen, Carlsbad, CA, USA) and plated at a density of 3 × 10^6^ live cells/60-mm plate. Neurospheres were dissociated every 4–5 days to facilitate cell growth. Cells were used between passages 10 and 20. All cells were confirmed to retain their differentiation capacity, mainly towards astrocytes, reducing the stem cell markers CD133 and Sox2, and increasing the astrocytic marker GFAP as described^12^. When indicated, GBM cells were incubated in the presence of 10% fetal calf serum, 50 ng/ml IL-6, 100–400 ng/ml Tocilizumab, 5–30 μM Ruxolitinib or cultured on glass surfaces coated with 10 μg/ml fibronectin (all from Sigma-Aldrich, St Louis, MO, USA). U937 cell line was obtained from ATCC (CRL-1593.2), cultured in RPMI 1640 (Invitrogen) with 10% fetal calf serum and maintained in culture for no more than ten passages after thawing. U937 cells were treated with 200 ng/ml Phorbol 12-myristate 13-acetate (PMA) either alone or with 5 μg/ml lipopolysaccharide (LPS) (both from Sigma-Aldrich). IL-6 secretion by U937 cells was quantified by using an ELISA kit (Quantikine ELISA kit form R&D Systems, Minneapolis, MN, USA).

All cells were tested for mycoplasma using the LookOut Mycoplasma qPCR Detection Kit (Sigma-Aldrich) within one week before the experimental work.

### Migration assay

The migratory capacity of GBM cell lines was determined by a modified Boyden chamber assay in 24-well plates (QCM 24-well colorimetric cell migration assay from Merck-Millipore, Darmstadt, Germany). GBM cell lines (500,000 cells) were placed in the upper compartment and following 24 h of incubation under the indicated conditions, cells that have migrated to the lower face of the membrane were fixed and stained according to the manufacturer’s instructions. Migration was determined by measuring absorbance at 560 nm in a spectrophotometer. Whenever indicated, U937 cell line (250,000 cells) were added to the lower compartment whereas GBM cells remained in the upper compartment.

### Immunofluorescence staining and analysis

Cells were incubated with antibodies against ODZ1^2^, followed by incubation with fluorescein isothiocyanate-conjugated goat anti-rabbit secondary antibodies (Jackson ImmunoResearch, Cambridgeshire, UK). Nuclei were visualized with 4′,6-diamino-2-phenylindole (DAPI) (Life Technologies, Paisley, UK).

### Gene expression analyses

The expression of individual genes was evaluated by qPCR on total cellular RNA as previously described^2^. cDNA was generated and amplified using the following primers: β-Actin (5′-GCGGGAAATCGTGCGTGACATT-3′ and 5′-GATGGAGTTGAAGGTAGTTTCGTG-3′), ODZ1 (5′-ACTCAAGAGATGGAATTCTGTG-3′ and 5′-CTTAGTGCATGGTCAGGTG-3′), Stat3 (5′-GGGTGGAGAAGGACATCAGC-3′ and 5′-GGTCTTCAGGTATGGGGCAG-3′), CCND1 (5′-CTGGCCATGAACTACCTGGA-3′ and 5′-GGGTCACAGTTGATCACTCTGG-3′) and G6PD (5′-ATCGACCACTACCTGGGCAA-3′ and 5′-TTCTGCATCACGTCCCGGA-3′). qPCR was performed in a 7000-sequence detection system (Life Technologies, Carlsbad, CA, USA).

Analysis of Stat3 target genes differentially expressed between GBM stem-like cells and FCS-treated (differentiated) GBM cells was performed in previous gene expression array data of our group^12^. The selection criteria was based on the fold-change value using a logFC cut-off of 1.5. The array data is deposited in a MIAME compliant database (GEO accession number GSE20736).

### Western blot analysis

Total protein from GBM cells were separated on 8% polyacrylamide gels and transferred to nitrocellulose. Blots were incubated with antibodies against pStat3-Ser727 (D8C2Z, Cell Signaling, Danvers, MA, USA), Stat3 (79D7, Cell Signaling) and GAPDH (sc-25778, Santa Cruz Biotechnology. Santa Cruz, CA, USA), followed by secondary anti-rabbit antibodies conjugated to horseradish peroxidase (sc-2357, Santa Cruz Biotechnology).

### Transfections, gene reporter assays and site-directed mutagenesis

We identified the ODZ1 promoter (Gene ID ENSG00000009694) and amplified a fragment of 1439 bp that included the transcription start site with primers 5′-ATTAGCCGGGCATGGTGGC-3′ and 5′-TGCAAGCAGTCCTGGAAGAG-3′ flanked by KpnI and XhoI sequences. The promoter was cloned into KpnI and XhoI sites within the cloning region of the pGL2-luciferase reporter vector (Promega, Madison, WI). GBM cells were cotransfected with 2 μg wild type or mutant promoter constructs, and 0.2 μg pRSV-β-gal by using nucleofection. Transfected cells were cultured for 48 h and cell extracts were prepared and analyzed for the relative luciferase activity by a dual-light reporter gene assays (Applied Biosystems, Foster City, CA). Results were normalized for transfection efficiency with values obtained with pRSV-β-gal. Site-directed mutagenesis of the Stat site in the ODZ1 promoter was performed by using the QuickChange site-directed mutagenesis kit (Agilent Technologies, Santa Cruz, CA) with the following primers (5′-GGATGTCTATAGATTGGTTTGCAAATACATATGGTGAAGAGC-3′ and 5′-GCTCTTCACCATATGTATTTGCAAACCAATCTATAGACATCC-3′). The modified promoter was sequenced to verify the mutation. When indicated, GBM cells were transfected with a dominant negative variant of Stat3 previously used by our group and others^28,29^ by using nucleofection and gene expression or promoter activity were analyzed after 48 h.

### Chromatin immunoprecipitation assay (ChIP)

ChIP assay was performed as previously described with some modifications^30^. Briefly, GBM cells were incubated with IL-6 for 24 h with or without Tocilizumab. Following crosslinking and cell lysis, chromatin was sonicated to generate fragments of about 1000 bp. Sonicated chromatin (10%) was kept as the input control material, the rest was incubated with antibodies against Stat3 (79D7, Cell Signaling) followed by incubation with Protein G Dynabeads (Invitrogen). All samples were treated with RNase A and Proteinase K and purified using ChIP DNA Clean and Concentrator (Zymo Research, Irvine, CA) before amplification. DNA was amplified using primers flanking the Stat site (5′-GTACATGATAGAGGAGCCACCA-3′ and 5′-GCAACTTTGCAACTTGGTCTT-3′) and primers of a region 9.5 kb upstream from the transcription start site (5′-GAAGGACCTCTTCAAGGAGA-3′ and 5′-GGATTGTCTTGGCTATACGG-3′) as a negative control. Data were calculated as the percentage of input DNA.

### Gene silencing

For ODZ1 knockdown experiments, we used two different shRNAs with sequences 5′-AATGGAGAATACGAGAAAGGACA-3′ and 5′-AAGACCGACATCTATGGACAGAA-3′ and a scrambled shRNA (Cat. No. P100042) as a negative control (all from Vigene Biosciences, Rockville, MD). Cells were transfected with shRNAs by nucleofection. Stat3 was silenced by transfecting GBM cells with a mixture of Stat3-specific (Cat. No. EHU122051) or negative control (Cat. No. EHUEGFP) siRNAs (MISSION esiRNA, Sigma-Aldrich) using Lipofectamine RNAiMAX (Invitrogen).

### Statistical analysis

All statistics were calculated with the SPSS statistical package (version 13.0). Data are presented as mean ± SD of three independent experiments. Differences between groups were tested for statistical significance using the unpaired 2-tailed Student’s t-test. The significance level was set at p < 0.05.
